# Abominations of the Back Yard

**Published:** 1885-10

**Authors:** Eben E. Rexford


### Abominations of the Back Yard.

Many back yards are abominations to the eye and nose. One finds in them all sorts of litter and refuse, from oyster cans to old boots. Here the slops of the kitchen are poured to increase the odors which ought to warn every thoughtful person of the malarial influence breeding there, to break out eventually in fevers or diphtheria. If any member of the family dies from one of these diseases his death is probably lamented as a "mysterious dispensation of Providence," but the minister would say, if he were to visit the back yard, that death was caused solely by a violation of hygienic laws. A very strong argument against a dirty back yard is the spirit of deception which it is apt to foster in the young members of the family, for it is a constant deceit to present a clean and attractive front yard to the gaze of the passers, while the back yard is not fit to be seen. Children should be taught to be clean for the sake of cleanliness, and not because outsiders are likely to criticise them. The best plan is to have a hogshead or large box fitted up in one corner of the yard, and make it a rule to throw into this old cans, boots, broken dishes, and all such rubbish, and when there is a great accumulation to bury or burn it. Do not allow anything to be thrown about. Have drains made to convey slops entirely from the house. Make good walks, and let the ground have a fine covering of grass, not weeds. Put up strong supports for the clothes line. Keep the fence in repair, and plant currant bushes near it. Set vines about the refuse barrel, and train them over it until it is hidden. If you have a receptacle for ashes, let it be something which can be shut up, not a row of old barrels to offend the eye, and give out a cloud of ashes every time the wind blows. Make it a rule to have the back yard at all times as clean as the front one.—*Eben E. Rexford, in American Agriculturist.*

